# SAW: Breaking Down Barriers between Art and Science

**DOI:** 10.1371/journal.pbio.0060211

**Published:** 2008-08-26

**Authors:** Anne Osbourn

## Abstract

The Science, Art and Writing initiative teaches young children that science and the arts are interconnected, taking advantage of the power of striking scientific images to stimulate children's interest in science and in understanding the natural world.

The path to specialization of knowledge starts early. By the time children leave primary school, they have already been taught to view subjects like biology, art, and social studies as unrelated disciplines rather than as interlocking pieces that together lay the foundation for a deeper understanding of the world. The divisions between science, the arts, and the humanities are reinforced in high school, where each subject is taught by a different instructor under pressure to “teach to the test,” a practice that further isolates subjects, stifles inquisitiveness, and quells creativity. By the time we become specialists as adults, our ability to recognize connections between disciplines tends to diminish even further—often at a price. If during this process, we lose the ability to communicate with other groups of specialists (for example, chemists, physicists, and mathematical modelers) or with those who have not had any formal science education beyond high school (which would include many taxpayers, politicians, and policy makers), if we become unresponsive to the needs of society, then our value to society becomes compromised. And the question arises: how do we break down these barriers or prevent them from becoming established in the first place, without compromising the standards of the different disciplines?

While high school students and adults often feel constrained by mental barriers, elementary school children have not yet been programmed into compartmentalized ways of thinking and have fewer inhibitions. They explore the world around them through personal adventure and discovery, inquiring, speculating, exploring, experimenting, and risk-taking. Children need these skills to realize their full potential and creativity as they grow into adulthood; scientists need them to break new ground. Without the ability or confidence to take risks as adults, whatever area we choose to specialize in, we are unlikely to make important contributions to our fields.

We all try to make sense of the world around us in our own ways, and although scientists and artists clearly approach their occupations in different ways, both depend on the ability to define a problem, note detail, enquire, and extract the essence of the problem in hand. Both require a combination of creativity and technical competence. The core of the scientific process entails critical thinking, the generation and testing of hypotheses, and the rigorous interpretation of data/observations, which in turn leads to further speculation, prediction, and testing. The general perception of artists seems to be that they engage in far more unrestrained interpretations of natural observations in order to understand the world around them, although I personally would be reluctant to suggest that these specialists are incapable of critical thought, experimental design, rigorous analysis, and iterative progressive development. These issues aside, both groups bring their own preconceived ideas, skill-sets, and perspectives to the problems they take on. Ultimately, they aim to pinpoint the “best truth possible” available to them at a given moment in time and to communicate this understanding clearly and succinctly to others for appraisal. As more perspectives are assimilated into these individual understandings, we can build up a composite, more refined and durable understanding.

One way to integrate science into the lives of students takes advantage of the natural curiosity of young children and the power of visual images to engage that curiosity to investigate science and the world. Stunning scientific images—particularly those that show something in an unusual way—act like magnets, attracting people of all ages and disciplines with their intriguing, non-threatening representations of natural phenomena. They awaken curiosity—a hunger to learn more. By using images from science as a starting point for scientific experimentation, art, and creative writing, the Science, Art and Writing (SAW) initiative (http://www.sawtrust.org/) breaks down barriers between science and the arts. Each SAW project has a scientific theme, supported by a collection of carefully selected visually striking scientific images. With this approach, children realize that science and the arts are interconnected—and they discover new and exciting ways of looking at the world.

Teachers can run SAW projects on their own; however, many of the projects that have taken place so far have involved teams of scientists, artists, and writers working with teachers to design and deliver projects. The teacher can work alongside the scientist to deliver the creative arts components of the project, or alternatively the school may like to invite a local poet and artist to join the SAW team. Scientists who would like to be involved in a SAW project can talk with a teacher and define a scientific theme that both would like to explore—perhaps a science curriculum topic or, even better, the scientist's own research area.

For example, my own research group works on plant-derived natural products, which two young scientists in my group, Sam Mugford (a postdoctoral researcher) and Melissa Dokarry (a Ph.D. student), chose as their SAW project theme. Sam and Melissa developed their one-day SAW project at Martham Primary School, Norfolk, United Kingdom, for a class of seven- to nine-year-olds, in close consultation with teacher Heather Delf. They selected the images shown in Figure 1 as a starting point to explore the theme, and introduced the children to the topic of plant-derived natural products, encouraging them to think about why plants might make chemicals that give them different scents, colors and flavors. The children and scientists had brought in a variety of different plant materials to investigate, and these specimens, along with the images, were used to stimulate discussion and speculation.

**Figure 1:**
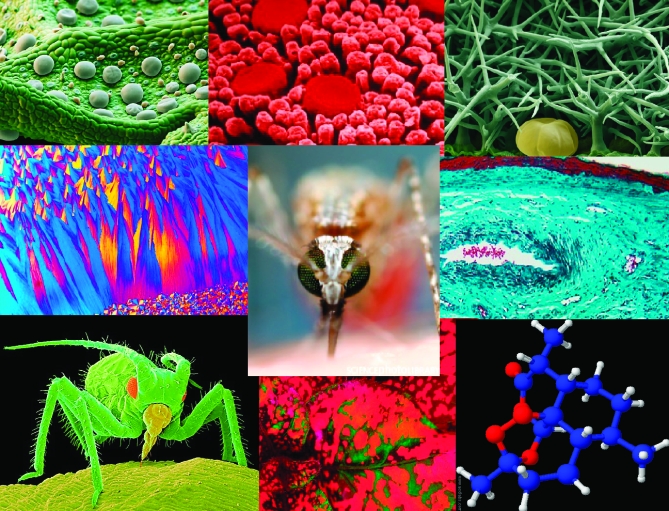
Stimulating Children's Interest in Science with Stunning Images Scientific images chosen by Sam Mugford and Melissa Dokarry, two young scientists in the Osbourn group, for a SAW project on plant-derived natural products. Top row, from left: The surface of a mint leaf showing trichomes, specialized cells that store the chemicals that give mint its smell and taste. (Image: Eye of Science/Science Photo Library.) A human tongue. (Image: Omikron/Science Photo Library.) The surface of a lavender leaf, showing the pumpkin-shaped gland that stores lavender oil. (Image: Eye of Science/Science Photo Library.) Middle row, from left: Polarized light micrograph of crystals of taxol, an important anti-cancer drug found in the bark of yew trees. (Image: Michael W. Davidson/Science Photo Library.) A mosquito sucking up blood. (Image: Sinclair Stammers/Science Photo Library.) The skin inside a nose. (Image: Astrid Kage/Science Photo Library.) Bottom row, from left: An aphid feeding on a leaf. (Image: Volker Steger/Science Photo Library.) Colored leaves. (Image: Steve Taylor/Science Photo Library.) Molecular model of artemisinin, a malaria drug extracted from wormwood (Artemisia annua). (Image: Sam Mugford.)

Sam and Melissa used the images of mint and lavender leaves under the microscope to highlight the specialized glands where the oils that give the flavors and scents are sequestered, and the children debated why these glands were necessary. Micrographs of the inside of the nose and taste buds provided a means of linking these compounds with the senses and of considering how the signals from these sensory organs are transmitted to the brain. The class then thought about how the colors, scents, and flavors of plants may attract or deter insects. This brief introduction to chemical ecology was supported by an image of an aphid feeding on a leaf. The class also discussed plants as a source of drugs and medicines. Sam and Melissa showed the children images of crystals of taxol (a cancer drug from the bark of yew trees) viewed under polarized light microscopy, and a molecular model of the malaria drug artemisinin during this part of the session. Through this introductory discussion, the children developed an awareness of the diversity of chemicals that plants produce. They also gained a better understanding of the properties and ecological significance of these chemicals and of their value for medical and other important uses.

The children then moved on to some practical science (see Figure 2), working with the collection of plants that the school and the scientists had supplied. The overall objective of the experimental work was to extract and analyze plant pigments. The children were encouraged to generate and test their own hypotheses—for example, do beetroot and red cabbage (which are both purple) contain the same or different pigments? Plant material (leaves or flowers) was ground up using pestles and mortars, filtered, and analyzed by paper chromatography. Acids (vinegar and lemon juice) and alkali (bicarbonate of soda solution) were also added to the plant extracts to test whether there was a pH-dependent change in color. Some children generated sets of “rainbow tubes” of graded colors by adding different amounts of acid or alkali to various samples. Wherever possible, children were encouraged to follow up on their ideas through investigations of their own design. Analysis of extracts often revealed mixtures of colors—for example, chlorophyll A and B were separated. There was discussion about what these pigments might do in plants. Towards the end of the practical session, Sam and Melissa introduced the structures and the names of some of the common plant pigments and described how these compounds would behave under the experimental procedures used. The children compared these properties with those of the compounds that they had extracted and correctly identified some of the compounds.

**Figure 2:**
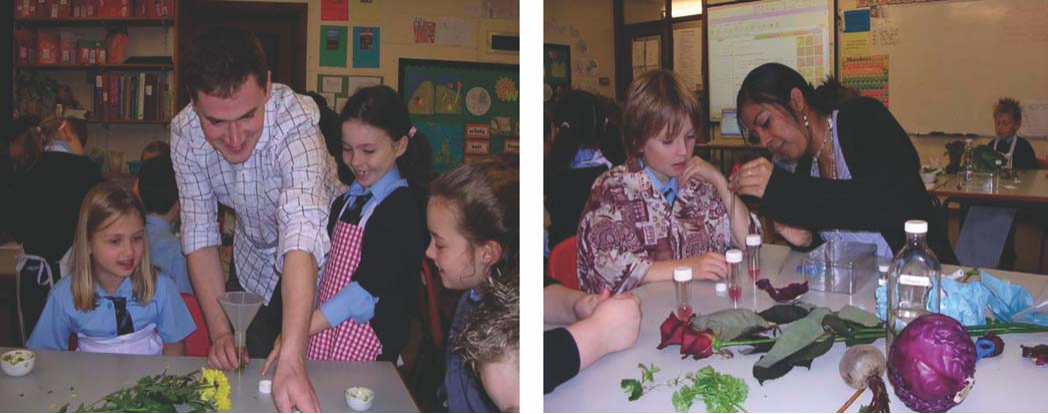
In the Classroom Sam Mugford (top left) and Melissa Dokkary (top right) showing 7- to 9-year olds at Martham Primary School how to extract and analyze pigments from plants.

After a break, the class moved on to poetry under the guidance of poets Mike O'Driscoll and Joe Mugford (Sam's brother). This session began with a brief introduction to poetry, with an emphasis on encouraging individual imagination and exploration over formal considerations. Parallels were drawn with science. Both scientists and artists closely observe and consider the available evidence and progress from there. During a discussion of the scientific images, the poets encouraged the children to use the images as creative springboards for writing poetry. The children were free to choose whichever image they liked and to explore it through poetry and/or to draw on other ideas inspired by the science session. Examples of both approaches are shown in Box 1. The freshness and imagination of children's approaches to poetry never ceases to amaze. It is clear from their poetry that the science had an impact on the children. After the class had written and revised their poems, the work was celebrated at the end of the session with readings. All of the children were proud of their work and keen to share it.

**Box 1. Natural Product Poetry**

In SAW workshops, children learn that both scientists and artists must closely observe and evaluate the natural world in their work. After a discussion of scientific images led by poets, children at Martham Primary School explored the scientific ideas represented in the images and discussed in class through poetry. Here is a small sample of the students' poetic interpretations of the day's lessons on plant-derived natural products.

**Science Nose**

As I crush the molecules with my fingers cells explode like a bomb.

Chemicals zoom upwards into my nostrils. Strange smells make my brain dance.

*Lloyd Sayer, Age 8*

**The Mosquito**

Bulging eyes with a multitude of visions form a complex compound kaleidoscope. Thorax as round as footballs, eyes pitted like golf balls.

Proboscis dangling loosely, like baubles on a Christmas tree.

*Kitty Hawkins, Age 8*

**Spreading Colours**

Colourful flames, the light looks alive, spreading slowly across the paper. Multi-coloured molecules, chemicals mixing together, sparkling in the light, dancing in the alcohol.

*Olivia Hesseltine, Age 7*

**A Crowd of People Looking Up to the Sky**

A crowd of people in a restaurant, waiting for their food.

Strawberries, tomatoes, spaghetti bolognaise.

Red icicles, like fruit sweets, squidgy and gooey like marshmallows and peas.

Bright pink coral under the sea.

People with their tongues hanging out, tasty, microscopic molecules drowning in a whirlpool of flavours.

*Darcie Lines, Age 8*

**Plant Science**

Leaves with millions of molecules fighting plant-eating bugs. Multi-coloured petals wave at the tips of long, thin stems. Bright yellow pollen is collected by busy, buzzing bees.

Spotted red ladybugs drift through the air. Bright petals spring forth like a Jack in a box. Lovely purple lavender fills the air with a colourful scent.

*Xena Dyball, Age 9*

After lunch, the final session was led by artist Chris Hann. Chris focused on the molecular model of the malaria drug artemisinin because he felt that the image lent itself to interpretation through sculpture. The aim was to encourage the children to interpret the image in their own way, to look for relationships between structures, colors, and forms, and to work in groups to make a three-dimensional model using colored polystyrene balls and straws (Figure 3).

**Figure 3:**
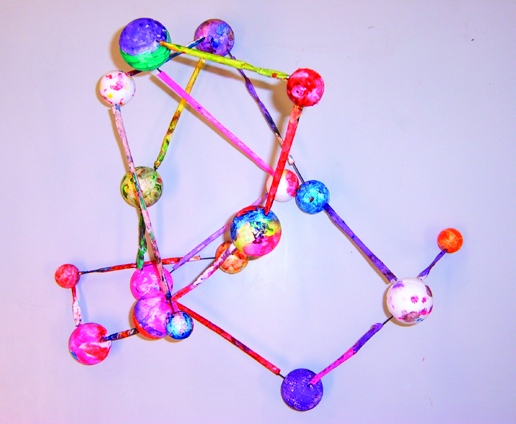
“Modeling” the Structure of the Malaria Drug Artemisinin

This union of science, art, and writing around a central scientific focus represents a powerful way of bringing science into the classroom. It breaks down barriers between science and the arts while demanding the highest standards of each individual discipline. The images provide a crucial anchor as the children move from science to the other disciplines. The children are totally immersed in the theme and thoroughly engaged in this intense learning experience. Using this multidisciplinary approach, SAW does more than just promote observation. It stimulates critical thinking, hypothesis-led scientific investigation, and creative exploration. Importantly, the exploration of the theme through different approaches, along with the central role of the images, enables children of different abilities and with different learning styles to find their own way into learning.

The project that Sam and Melissa designed was one of 15 projects run by scientists at the John Innes Centre and the Institute of Food Research in Norwich, UK, funded by an outreach grant from the Biotechnology and Biological Sciences Research Council. The 15 projects were designed around different facets of scientific research carried out at these two institutes, and included projects on starch, fractals, DNA, emulsions, Streptomyces, Salmonella, and other research themes. The scientists were given advice on how to set up and run their projects. Once they had found a school to work with and had defined their theme and images, they were introduced to the poets and artists who made up the rest of the SAW team. Once the poets and artists had been briefed on the theme and given the scientific images to work with, they decided how best to use the theme and the images to design high-quality creative adventures. The poets and artists are not expected to be scientists, in the same way that the scientists are not expected to be poets or artists. The scientists, poets, and artists must be true to their own respective disciplines. In this way the highest standards are maintained across the disciplines.

In the 21st century, schools are increasingly being viewed as learning communities—everybody learning together, both adults and children. Priorities include personalized learning, social development, creative and critical thinking, relevant learning and links to the real world, transferable cross-curricular skills, challenge, and enterprise. Community participation and the involvement of experts are important components of this equation. The coming together of artists and scientists to work with teachers and children in cross-curricular activities that explore science and the world is therefore very timely. The celebration of the fruits of these activities through sharing within the school and the wider community provides an extra dimension to the whole process and makes science accessible to all.

## Abbreviations

SAW: Science, Art and Writing

## References

[pbio-0060211-b001] Osbourn A, Pirrie J, Nicholson J, Holbeck K, Hogden S (2005). See Saw. An anthology of poetry and artwork around science by children from Rockland St. Mary County Primary School and Framingham Earl High School, working with Matthew Sweeney and Jill Pirrie.

